# Clinical translation potential of self‐inspired live‐cell super‐resolution microscopy

**DOI:** 10.1002/ctm2.70390

**Published:** 2025-07-01

**Authors:** Liying Qu, Jingyang Zhu, Xiangyan Ding, Haoyu Li, Weisong Zhao

**Affiliations:** ^1^ Innovation Photonics and Imaging Center, School of Instrumentation Science and Engineering, State Key Laboratory of Matter Behaviors in Space Environment, Frontier Science Center for Interaction Between Space Environment and Matter Harbin Institute of Technology Harbin China

**Keywords:** clinical translation, live‐cell imaging, self‐supervised learning, super‐resolution microscopy

## 1 INTRODUCTION

Super‐resolution microscopy (SRM) has transformed our capacity to visualise subcellular structures,^1^ offering unparalleled detail for biomedical research and clinical diagnostics.^2^, ^3^, ^4^, ^5^ However, the inherent photon budget constraints in live‐cell imaging have long impeded the full realisation of these techniques, particularly when high spatiotemporal resolution is crucial for tracking dynamic biological processes.^6^ To address this fundamental challenge, in our recent work published in *Nature Methods*, we introduced Self‐inspired Noise2Noise (SN2N),^7^ a deep learning framework that substantially enhances photon efficiency in live‐cell SRM by one to two orders of magnitude. Here, we discuss the profound clinical translational potential we believe this innovation unlocks.

## 2 TECHNICAL INNOVATION WITH CLINICAL SIGNIFICANCE

A key innovation of SN2N, as we designed it, is its capacity for robust denoising without the need for clean reference images or paired noisy training data. Crucially, its ability to train effectively from a single noisy frame, by leveraging inherent spatial redundancy in super‐resolution images, offers a paradigm shift. For clinical research, this translates directly to markedly improved signal‐to‐noise ratios under low‐illumination conditions. This, in turn, enables the adoption of gentler imaging protocols, thereby minimising phototoxicity and photobleaching—persistent challenges in live‐cell SRM that we aimed to overcome.

This advance is particularly pertinent for studies involving patient‐derived primary cells or biopsy tissues. Here, long‐term observation of disease progression, drug efficacy and cellular dynamics can be achieved with minimal sample perturbation. Traditional SRM approaches often necessitate intense illumination, risking cellular stress, altered physiological states or even cell death.^8^, ^9^ Such effects can compromise observational validity, a critical concern for sensitive samples such as cancer biopsies or stem cells. Our development of SN2N seeks to mitigate these limitations.

## 3 CLINICAL APPLICATIONS OF ENHANCED 5D IMAGING

**FIGURE 1:**
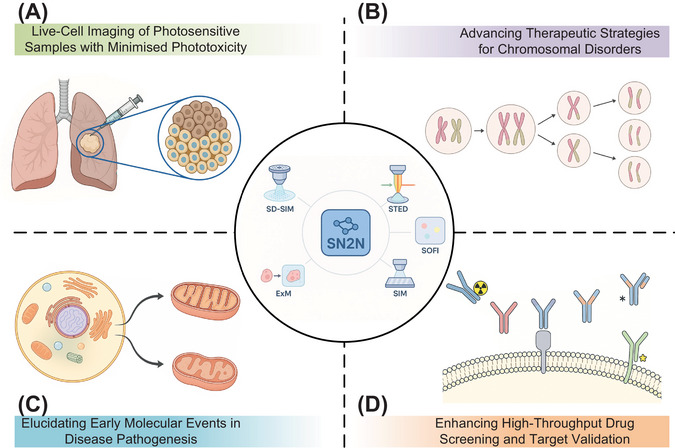
Clinical translational potential of the Self‐inspired Noise2Noise (SN2N)‐enhanced live‐cell super‐resolution microscopy. The SN2N framework (centre) is compatible with various super‐resolution microscopy modalities (e.g., spinning‐disc confocal‐based structured illumination microscopy [SD‐SIM], stimulated emission depletion [STED], SR optical fluctuation imaging reconstruction [SOFI], structured illumination microscopy [SIM], expansion microscopy [ExM]). By improving photon efficiency by one to two orders of magnitude, SN2N enables high‐fidelity imaging under reduced illumination, minimising phototoxicity and photobleaching. This facilitates diverse clinical research applications, including: (A) Live‐cell imaging of photosensitive samples (e.g., patient‐derived biopsies) with minimised phototoxicity for long‐term observation of disease progression and therapeutic responses. (B) Enabling extended, high‐resolution observation of dynamic processes like mitosis, offering insights into chromosomal instability mechanisms relevant to therapeutic strategies for chromosomal disorders. (C) Visualising subtle morphological and dynamic alterations for elucidating early diagnostic markers in diseases. (D) Improving high‐throughput drug screening and target validation by allowing accurate assessment of compound effects on cellular architecture and biomolecular interactions with minimal phototoxic perturbation. Schematics were created with BioRender.com.

SN2N's optimisation for 5D imaging (xyz‐colour‐time) represents an achievement of our framework. We demonstrated its application in facilitating the first complete observation of mitosis in live cells over a 3‐h period, maintaining <100 nm spatial resolution. Given that mitosis is highly susceptible to phototoxicity, where conventional imaging often induces cell cycle arrest, chromosomal missegregation or apoptosis,^10^ this capability to observe the entire process with such fidelity is noteworthy. The reduced phototoxicity afforded by SN2N offers a window into cell division mechanisms and the development of anti‐mitotic therapies, pertinent for understanding chromosomal instability disorders and refining targeted cell cycle interventions (Figure 1).

## 4 MULTI‐MODAL COMPATIBILITY AND CLINICAL RESEARCH APPLICATIONS

SN2N exhibits broad compatibility with diverse imaging modalities, including spinning‐disc confocal‐based structured illumination microscopy (SD‐SIM), stimulated emission depletion (STED) microscopy, SR optical fluctuation imaging reconstruction (SOFI), structured illumination microscopy (SIM) and expansion microscopy (ExM). This versatility facilitates its integration into existing clinical research workflows without necessitating costly hardware modifications.

In neurodegenerative disease research, (e.g., Alzheimer's and Parkinson's^11^), where early mitochondrial alterations are critical pathogenic events, SN2N‐enhanced STED microscopy allows prolonged, high‐resolution observation of dynamic mitochondrial cristae remodelling and fusion‐fission events with minimal phototoxicity. This capability, we believe, offers new avenues for elucidating disease mechanisms, identifying early biomarkers and assessing therapeutic efficacy. The synergy of SN2N with ExM also improves super‐resolution imaging of expanded tissue biopsies, presenting substantial translational opportunities. By enhancing image clarity, it provides richer molecular‐level detail crucial for precise pathological assessment,^12^ for instance, enabling clearer visualisation of subtle podocyte changes in nephrology indicative of early glomerular injury.^13^ Furthermore, SN2N benefits computational SRM techniques like SIM and SOFI, which are prone to artefacts from raw data noise and statistical uncertainties in reconstruction. SN2N mitigates these issues by reducing noise‐induced errors through cleaner input data and by suppressing artefacts from statistical fluctuations and instabilities via its self‐supervised learning. This yields more robust and faithful structural representations, crucial for reliable quantitative analysis of subtle cellular changes.

## 5 APPLICATIONS IN DRUG DISCOVERY AND DEVELOPMENT

For drug discovery and development, SN2N provides distinct advantages in high‐content screening and target validation. By enabling high‐quality imaging under lower phototoxicity, it allows for a more accurate and prolonged assessment of compound effects, mitigating issues of cellular stress and toxicity often encountered with conventional high‐illumination approaches.^14^ For instance, in cancer drug screening, SN2N permits detailed observation of subtle, yet critical, drug‐induced cellular alterations (e.g., autophagosome biogenesis,^15^ mitochondrial membrane potential^16^)—which serve as key indicators of drug efficacy, and is valuable for characterising compounds with delayed‐action or cumulative effects.

## 6 POST‐TRAINING DEPLOYMENT POTENTIAL

A key feature of SN2N is its post‐training deployment flexibility and scalability. Once trained, the network can be readily deployed across diverse clinical and research settings, often without extensive local computational infrastructure. This ‘train once, deploy anywhere’ paradigm makes SN2N advantageous for resource‐constrained environments.

In multicentre clinical trials, SN2N can be centrally trained and subsequently distributed, ensuring consistency in image quality and analytical outcomes. Such standardisation is pivotal for robust biomarker evaluation.^17^ For instance, in multicentre oncology trials, SN2N could standardise subcellular analyses of live tissue biopsies, enhancing data comparability and reliability across sites. Furthermore, trained SN2N models can be integrated into existing clinical pathology workflows without requiring pathologists to master deep learning. This seamless adoption enables routine pathological examinations to benefit from super‐resolution imaging, without increasing operational complexity or diagnostic timelines.

## 7 INTEGRATION WITH AUTOMATED SEGMENTATION AND FUTURE OUTLOOK

We are currently extending the SN2N framework to incorporate automated segmentation capabilities. By leveraging deep learning for accurate identification and classification of subcellular structures, this integration can accelerate data analysis pipelines, mitigate subjective bias and enable high‐throughput processing of large‐scale datasets. Incorporating spatiotemporal information, SN2N‐assisted organelle segmentation can reveal subtle shifts in organelle morphology and dynamics, crucial for screening therapeutics targeting specific organelle dysfunctions.

## 8 CONCLUSION

SN2N represents a key advance we have made in computational imaging, addressing the fundamental photon budget limitations of live‐cell SRM. By enabling high‐quality imaging with reduced illumination, this technology enables new possibilities for observing cellular processes relevant to disease research, drug development and clinical diagnostics. Its ability to deliver notable performance gains without expensive hardware upgrades makes SN2N a practical tool for translating insights from fundamental science into clinical application. Nonetheless, widespread clinical translation requires surmounting challenges in broad validation and seamless workflow integration. As we continue to optimise SN2N and pursue its clinical validation, we anticipate its emergence as a tool to advance precision medicine and personalised treatment, ultimately aiming for tangible patient benefits and potentially reshaping our understanding and treatment of diseases.

## AUTHOR CONTRIBUTIONS

W.Z. conceived and supervised the work. L.Q. wrote the manuscript. J.Z. prepared the figures and, along with X.D. and H.L., helped shape the manuscript. All authors participated in the discussions, revised the manuscript, and approved the final version.

## ETHICS STATEMENT

Not applicable.
